# Possible Involvement of Mitochondrial Dysfunction and Oxidative Stress in a Cellular Model of NAFLD Progression Induced by Benzo[a]pyrene/Ethanol CoExposure

**DOI:** 10.1155/2018/4396403

**Published:** 2018-07-26

**Authors:** Simon Bucher, Dounia Le Guillou, Julien Allard, Grégory Pinon, Karima Begriche, Arnaud Tête, Odile Sergent, Dominique Lagadic-Gossmann, Bernard Fromenty

**Affiliations:** ^1^Univ Rennes, Inserm, Inra, Institut NUMECAN (Nutrition Metabolisms and Cancer)–UMR_S 1241, and UMR_A 1341, 35000 Rennes, France; ^2^Univ Rennes, Inserm, EHESP, and Irset (Institut de recherche en santé, environnement et travail)–UMR_S 1085, 35000 Rennes, France

## Abstract

Exposure to xenobiotics could favor the transition of nonalcoholic fatty liver (NAFL) to nonalcoholic steatohepatitis in obese patients. Recently, we showed in different models of NAFL that benzo[a]pyrene (B[a]P) and ethanol coexposure induced a steatohepatitis-like state. One model was HepaRG cells incubated with stearate and oleate for 2 weeks. In the present study, we wished to determine in this model whether mitochondrial dysfunction and reactive oxygen species (ROS) overproduction could be involved in the occurrence of this steatohepatitis-like state. CRISPR/Cas9-modified cells were also used to specify the role of aryl hydrocarbon receptor (AhR), which is potently activated by B[a]P. Thus, nonsteatotic and steatotic HepaRG cells were treated with B[a]P, ethanol, or both molecules for 2 weeks. B[a]P/ethanol coexposure reduced mitochondrial respiratory chain activity, mitochondrial respiration, and mitochondrial DNA levels and induced ROS overproduction in steatotic HepaRG cells. These deleterious effects were less marked or absent in steatotic cells treated with B[a]P alone or ethanol alone and in nonsteatotic cells treated with B[a]P/ethanol. Our study also disclosed that B[a]P/ethanol-induced impairment of mitochondrial respiration was dependent on AhR activation. Hence, mitochondrial dysfunction and ROS generation could explain the occurrence of a steatohepatitis-like state in steatotic HepaRG cells exposed to B[a]P and ethanol.

## 1. Introduction

Nonalcoholic fatty liver disease (NAFLD) refers to the large spectrum of hepatic lesions linked to obesity including nonalcoholic fatty liver (NAFL), nonalcoholic steatohepatitis (NASH), and cirrhosis. Most obese individuals present NAFL (also referred to as hepatic steatosis), which is characterized by the accumulation of triglycerides as large cytoplasmic vacuoles. Although NAFL is benign in the short term, it can progress in the long term to NASH in up to one third of patients [1, 2]. NASH is characterized not only by fat accumulation but also by necroinflammation, fibrosis, and the presence of apoptotic hepatocytes [1, 3]. Notably, NASH can also evolve in some patients to cirrhosis and hepatocellular carcinoma (HCC) [1, 2]. Several genetic polymorphisms could explain, at least in part, why NAFL progresses to NASH in some obese patients [4, 5]. Nongenetic factors might also play a role such as exposure to different types of xenobiotics including drugs, alcohol, and environmental contaminants [6–9].

The polycyclic aromatic hydrocarbon benzo[a]pyrene (B[a]P) is an environmental contaminant present in diesel exhaust particles, cigarette smoke, and grilled and smoked food [10, 11]. B[a]P is a potent activator of the transcription factor aryl hydrocarbon receptor (AhR), which regulates the expression of different xenobiotic metabolizing enzymes (XMEs) including cytochrome P450 1A1 (CYP1A1) and CYP1A2 [12, 13]. Recently, we showed in three different models of NAFL that B[a]P and ethanol coexposure induced the appearance of a steatohepatitis-like state characterized by hepatocyte demise and increased expression of several inflammation markers such as interleukin 1*β* (IL1*β*), IL6, tumor necrosis factor *α* (TNF*α*), and C-reactive protein (CRP) [14]. Importantly, these different deleterious effects of B[a]P and ethanol coexposure were overall stronger than the respective effects observed for each compound [14]. The models of NAFL used in that study included two *in vitro* models, respectively, human HepaRG cells and hybrid human/rat WIF-B9 cells overloaded with fatty acids (FAs) and an *in vivo* model, zebrafish larvae fed daily with a high-fat diet (HFD). Notably, the steatohepatitis-like state in HepaRG cells was characterized by a strong reduction of the expression of numerous XMEs, which was associated with lesser B[a]P detoxification and higher formation of B[a]P trans-7,8-dihydrodiol, a precursor of several toxic B[a]P metabolites [14].

In the present study, we wished to determine in the HepaRG cell model of NAFL whether mitochondrial dysfunction and reactive oxygen species (ROS) production could be involved in the occurrence of this steatohepatitis-like state. Indeed, mitochondrial dysfunction and ROS generation are key events in the pathological transition of NAFL to NASH [4, 15]. We also wished to specify the role of AhR in these deleterious events since this transcription factor plays a significant role in B[a]P toxicity [16, 17].

## 2. Materials and Methods

### 2.1. Chemicals and Reagents

B[a]P, ethoxyresorufin, dimethyl sulfoxide (DMSO), oleic acid, stearic acid, 4-hydroxy-TEMPO (Tempol), Hank's balanced salt solution (HBSS) (with calcium and magnesium, without phenol red), and insulin were purchased from Sigma-Aldrich (Saint-Quentin-Fallavier, France). [U-^14^C]-Palmitic acid was supplied by Perkin Elmer (Villebon-sur-Yvette, France). William's E medium, glutamine, Hoechst 33342 and Nile red dyes, MitoSOX Red, dichlorodihydrofluorescein diacetate (H_2_DCFDA), penicillin, streptomycin, and the bicinchoninic acid kit for protein quantification were from Thermo Fisher Scientific (Courtabœuf, France). Fetal bovine serum (FBS) was supplied by Lonza (Levallois-Perret, France). Hydrocortisone hemisuccinate was purchased from SERB Laboratories (Paris, France). Protease and phosphatase inhibitors were supplied by Roche Diagnostics (Indianapolis, IN). Ethanol was purchased from VWR Chemicals (Fontenay-sous-Bois, France). 2-(1H-Indol-3-ylcarbonyl)-4-thiazolecarboxylic acid methyl ester (ITE) was from Bio-Techne (Lille, France). AhR antibody (D5S6H) was provided by Cell Signaling (Danvers, MA). *β*-Actin antibody (MA1115) and aldolase B (CS12063) antibody were from Santa Cruz Biotechnology (Dallas, TX). Cytochrome P450 3A4 (CYP3A4) antibody (AB1254) and CYP2E1 antibody (AB1252) were purchased from EMD Millipore (Billerica, MA) and albumin antibody (SKU114002) from Kent Laboratories (Bellingham, WA).

### 2.2. Cell Culture and Treatments

Native HepaRG cells were cultured as previously described [18]. Briefly, HepaRG cells were seeded at a density of 2.6 × 10^4^ cells/cm^2^ and were first incubated in William's E medium supplemented with 10% FBS, 100 units/ml penicillin, 100 *μ*g/ml streptomycin, 2 mM glutamine, 5 *μ*g/ml insulin, and 50 *μ*M hydrocortisone hemisuccinate. After 2 weeks, cell differentiation was induced by culturing HepaRG cells in the same medium supplemented with 2% DMSO for 2 additional weeks. Induction of steatosis with stearic and oleic acids (150 *μ*M each) and treatments with B[a]P (2.5 *μ*M) and ethanol (25 mM) were carried out exactly as described in our recent study [14]. Indeed, differentiated HepaRG cells were first incubated for 2 days in a medium containing 5% FBS and 1% DMSO with or without a mixture of stearic and oleic acids. Next, HepaRG cells cultured in the same medium were treated every 2 or 3 days for 2 weeks with or without B[a]P and/or ethanol in association or not with the fatty acid mixture [14]. A scheme representing the protocol of cell culture and treatments is provided in Supplementary Figure 1. All 14-day investigations were performed 24 h after the last treatment. HepaRG cells were used at passages 10 to 16. In some experiments, HepaRG cells were treated or not with ITE (25 *μ*M) or Tempol (25 *μ*M) for 2 weeks. These compounds were added in the medium every 2 or 3 days concomitantly to the other treatments. Some investigations on mitochondrial respiration using the Seahorse technology (see below) were also performed in differentiated HepaRG cells treated once with B[a]P (2.5 or 5 *μ*M) or ITE (5 or 10 *μ*M). In this case, investigations were performed immediately after the treatment. The protocol of drug injection in the Seahorse analyzer and the limited solubility of ITE did not allow us to study concentrations above 10 *μ*M.

### 2.3. Nile Red Staining and Cellular Triglyceride Assay

The Nile red dye allows the staining of neutral lipids within cells [19]. For Nile red staining, cells were washed with warm phosphate-buffered saline (PBS), fixed with 4% paraformaldehyde for 20 min at room temperature, and then washed three times with cold PBS. After paraformaldehyde fixation, cells were incubated with 0.05 *μ*g/ml Nile red in PBS for 30 min at room temperature and then washed once with PBS. Nuclei were labelled with 10 *μ*g/ml Hoechst 33342 dye, and cells were observed with an ImageXpress Micro Confocal High-Content Imaging System (Molecular Devices, Berkshire, UK). Excitation/emission wavelengths were 377/447 and 351/593 nm, respectively, for Hoechst 33342 and Nile red. Triglycerides were measured with the colorimetric kit purchased from BioVision (Milpitas, CA), according to the manufacturer's recommendations.

### 2.4. Isolation of RNA and Gene Expression Assessment

Total RNA was extracted from about 6 × 10^5^ HepaRG cells with the NucleoSpin RNA isolation system purchased from Macherey-Nagel (Hœrdt, France), which included a DNase treatment step. RNAs were reverse-transcribed into cDNAs using the High-Capacity cDNA Reverse Transcription kit from Applied Biosystems (Woolston, UK). mRNA gene expression was then assessed by quantitative PCR analysis (qPCR) using the SYBR Green PCR Master Mix (Applied Biosystems) and an Applied Biosystems 7900HT Fast Real-Time PCR System (Applied Biosystems). Expression of the TATA-box binding protein (TBP) gene was used as reference, and the 2^–ΔΔCt^ method was employed to express the relative expression of each selected gene. Sequences of the primers used to assess gene expression are presented in Supplementary Table 1.

### 2.5. Measurement of Mitochondrial Fatty Acid Oxidation, Mitochondrial Respiration, and Activity of Respiratory Chain Complexes I, II, and IV

Mitochondrial fatty acid oxidation (FAO) was measured in adherent HepaRG cells using [U-^14^C] palmitic acid (0.05 *μ*Ci/ml), as previously described [20]. Results were normalized to the total protein content determined by the bicinchoninic acid method. Results were expressed in comparison to nonsteatotic untreated cells.

Mitochondrial respiration in the presence of L-glutamine (2 mM), glucose (10 mM), and pyruvate (1 mM) was measured in adherent treated HepaRG cells using an Agilent Seahorse XFe24 analyzer and XF Cell Mito Stress Test kits from Agilent (Santa Clara, CA), according to the manufacturer's instructions. For the experiments carried out in differentiated HepaRG cells treated once with B[a]P and ITE, these compounds were directly injected by the Seahorse device in the medium just before the start of the measurement of mitochondrial respiration with the XF Cell Mito Stress Test kit. Oxygen consumption rates provided by the Seahorse Mito Stress Test profile and corresponding to basal respiration, maximal respiration (induced by the OXPHOS uncoupler FCCP), ATP production, and proton leak were normalized in each well to the number of cells estimated by the fluorescence intensity of the Hoechst 33342 dye (10 *μ*g/ml) using the POLARstar Omega microplate reader from BMG Labtech (Ortenberg, Germany) with excitation/emission wavelengths of 355/460 nm.

Mitochondrial respiratory chain (MRC) complex I and IV activities were measured, respectively, with about 25 and 15 *μ*g of proteins with the Complex I and Complex IV Enzyme Activity Dipstick Assay kits from Abcam (Paris, France), according to the manufacturer's instructions. Briefly, complex I or IV was first immunocaptured and immunoprecipitated in active form on the dipstick that consists of a nitrocellulose membrane to which an anti-complex I or anti-complex IV monoclonal antibody is attached. The dipstick was then immersed in a buffer containing the appropriate substrates and electron acceptors (NADH and nitrotetrazolium blue for complex I or reduced cytochrome *c* and diaminobenzidine tetrachloride for complex IV), and activities were then measured immunochromatographically using a Hamamatsu MS1000 Dipstick Reader (Abcam). Results were expressed in comparison to nonsteatotic untreated cells. Activity of the MRC complex II, also referred to as succinate dehydrogenase (SDH), was measured with about 60 *μ*g of proteins using the Complex II Enzyme Activity Microplate Assay kit from Abcam, according to the manufacturer's recommendations. Results were expressed in comparison to nonsteatotic untreated cells.

### 2.6. Assessment of Mitochondrial DNA Levels

Total DNA was extracted from about 6 × 10^5^ HepaRG cells using the Blood and Cell Culture DNA Mini Kit from Qiagen (Les Ulis, France). The relative content of mitochondrial DNA (mtDNA) was then assessed by qPCR as described above. The primers used to amplify a portion of the mtDNA gene encoding the cytochrome *c* oxidase subunit 1 (MT-CO1) were 5′-TACGTTGTAGCCCACTTCCACT-3′ (forward) and 5′-AGTAACGTCGGGGCATTCCG-3′ (reverse). For normalization of mtDNA content, a portion of the nuclear DNA gene encoding the ribosomal protein S6 (RPS6) was amplified by using the forward primer 5′-TGATGTCCGCCAGTATGTTG-3′ and the reverse primer 5′-TCTTGGTACGCTGCTTCTTC-3′. The 2^–ΔΔCt^ method was then employed to assess the relative mtDNA levels. Results were expressed in comparison to nonsteatotic or steatotic untreated cells, as appropriate.

### 2.7. Assessment of ROS Generation

ROS generation was assessed using the H_2_DCFDA and MitoSOX Red dyes, allowing the detection of cellular hydrogen peroxide and mitochondrial superoxide anion, respectively [21, 22]. Cells were washed with warm HBSS and then incubated for 25 min at 37°C with 2 *μ*M H_2_DCFDA or 5 *μ*M MitoSOX Red, respectively, diluted in HBSS. Cells were then gently washed once with warm HBSS buffer. Fluorescence intensity of living cells was determined by spectrofluorimetry using the POLARstar Omega microplate reader (BMG Labtech) with excitation/emission wavelengths of 485/520 nm for H_2_DCFDA and 520/590 nm for MitoSOX Red. Fluorescence intensity values were normalized to the total protein content. Results were expressed in comparison to nonsteatotic or steatotic untreated cells, as appropriate.

### 2.8. Assessment of Caspase-3 Activity and LDH Release

Caspase-3 activity was measured by using the EnzChek™ Caspase-3 Assay kit purchased from Invitrogen Molecular Probe, using the manufacturer's instructions. Briefly, about 6 × 10^5^ cells were washed with PBS and then lysed in 65 *μ*l of the kit lysis buffer. The caspase-3 substrate Z-DEVD-AMC was added to a final concentration of 200 *μ*M in 50 *μ*l of the kit reaction buffer, which was then mixed to an equivalent volume of cell lysate. Fluoromethylcoumarin fluorescence, resulting from the caspase-3-mediated cleavage of Z-DEVD-AMC, was detected using the POLARstar Omega microplate reader (BMG Labtech) with excitation/emission wavelengths of 350/450 nm with a 45-min kinetics. The remaining cell lysate was used to normalize the fluorescence values to the total protein content. Results were expressed in comparison to nonsteatotic or steatotic untreated cells, as appropriate. Lactate dehydrogenase (LDH) release in the culture medium was used as an indicator of cell necrosis, as previously reported [23, 24]. To this end, LDH activity was measured in culture medium aliquots using the Lactate Dehydrogenase Assay Kit purchased from Abcam, according to the manufacturer's recommendations. Results were expressed in comparison to nonsteatotic untreated cells.

### 2.9. Generation of AhR-Deficient HepaRG Cells

The gene encoding the aryl hydrocarbon receptor (*AHR*) was targeted in HepaRG cells using the CRISPR/Cas9 system in order to introduce a frame shift into the coding sequence, as described previously [25], with some modifications. Briefly, exon 2 *AHR*-targeting gRNA sequence (5′-AAGTCGGTCTCTATGCCGCT-3′), designed using the CRISPOR web tool (http://crispor.tefor.net), was inserted into the linearized GeneArt CRISPR Nuclease Vector Kit OFP Reporter system (Thermo Fischer Scientific) using two partially annealing oligonucleotides with cohesive ends (5′-AAGTCGGTCTCTATGCCGCTGTTTT-3′ and 5′-AGCGGCATAGAGACCGACTTCGGTG-3′; Eurogentec, Liège, Belgium). The ligated plasmid was used to transform *E. coli* bacteria (NEB 10-beta Competent *E. coli*; New England Biolabs, Evry, France). The transformed bacteria were then isolated and selected on ampicillin-containing Luria-Bertani (LB) agar plates. Ten colonies were picked, and plasmid was purified using the NucleoSpin Plasmid MiniPrep kit from Macherey-Nagel, followed by sequencing of the inserted fragments using the U6 forward primer (supplied with the GenArt kit) by the Sanger method (GATC Biotech, Constance, Germany). Bacteria transformed with the plasmid containing the appropriate exon 2 *AHR*-targeting gRNA sequence were amplified, and plasmids were purified using the endotoxin-free NucleoBond Xtra Maxi Plus EF kit from Macherey-Nagel. HepaRG cells (about 10^6^ cells/well in 6-well plates) were then transfected using the SYN1-liposome (SynNanoVect Biogenouest core facility, Rennes, France) with a plasmid/lipid ratio of 20 *μ*g/60 *μ*l prepared in Opti-MEM medium (Thermo Fischer Scientific). Two days later, cells were trypsinized and sorted using a FACSAria flow cytometer (BIOSIT core facility, Rennes, France). Orange fluorescent protein- (OFP-) positive cells were collected and seeded for amplification. The detection of mutations was performed with the T7 endonuclease I enzyme using the EnGen Mutation Detection kit from New England Biolabs, in accordance with the manufacturer's instructions. A clonal selection was performed by seeding 1 cell/well in a 96-well plate. After growth and expansion, the cell clone of interest (henceforth referred to as mut*AhR*-HepaRG cells) was selected among about 60 different clones on four parameters: (1) cell morphology, (2) AhR mRNA and protein expression, (3) EROD activity after a 2-day treatment with the potent AhR activator ITE, and (4) mRNA and protein expression of aldolase B, albumin, CYP3A4, and CYP2E1, which are bona fide markers of hepatocellular differentiation [26].

### 2.10. Western Blot Analysis

Cells were harvested, washed twice with ice-cold PBS, and lysed in a RIPA buffer (150 mM NaCl, 50 mM Tris-HCl pH 7.4, 0.25% sodium deoxycholate, 0.1% sodium dodecyl sulfate, 1 mM EDTA, and 1% NP-40) supplemented with protease and phosphatase inhibitors. About 25 *μ*g of proteins was then separated by electrophoresis on NuPAGE 4–12% gradient Bis-Tris gels (Thermo Fischer Scientific) and transferred to 0.2 *μ*m nitrocellulose membranes (Bio-Rad, Les Ulis, France), which were saturated for 2 h at room temperature with 5% bovine serum albumin (BSA) or 5% skimmed dry milk (according to antibodies and manufacturers' recommendations), in Tris-buffered saline (TBS) containing 0.1% Tween 20 (TBS-T). Proteins were then immunoblotted with antibodies against CYP3A4, CYP2E1, AhR, albumin, aldolase B, and *β*-actin (used as loading control). The dilution used for all antibodies was 1 : 1000 except for *β*-actin (1 : 5000). Finally, blots were incubated with appropriate secondary antibodies, and protein bands were revealed by enhanced chemiluminescence with the Fusion FX7 Spectra system (Vilber Lourmat, Eberhardzell, Germany).

### 2.11. Measurement of CYP2E1, CYP3A4, and EROD Activity

CYP2E1 activity was measured by determining the generation of chlorzoxazone O-glucuronide from chlorzoxazone with a high-performance liquid (HPLC) method [27]. Briefly, HepaRG cells were incubated for 6 h at 37°C in phenol red-free and DMSO-free William's E medium containing 300 *μ*M chlorzoxazone. At the end of the incubation, aliquots of culture medium were collected and chlorzoxazone O-glucuronide was measured by HPLC analysis with UV detection at 287 nm. CYP2E1 activity was expressed as pmol/mg protein/h. CYP3A4 activity was also measured using a HPLC method [18]. Briefly, HepaRG cells were incubated for 2 h at 37°C in phenol red-free William's E medium containing 0.1% DMSO and 200 *μ*M testosterone. At the end of the incubation, aliquots of culture medium were collected and 6*β*-hydroxytestosterone was then measured by HPLC analysis with UV detection at 245 nm. CYP3A4 activity was expressed as nmol/mg protein/h. Ethoxyresorufin-O-deethylase (EROD) activity was used to measure CYP1A1, CYP1A2, and CYP1B1 activities [28, 29]. Resorufin formation was monitored using the POLARstar Omega microplate reader (BMG Labtech), with excitation/emission wavelengths of 520/590 nm. Reaction rates were determined under linear conditions and normalized to the total protein amount.

### 2.12. Assessment of ATP Levels

Cellular ATP levels were assessed using the CellTiter-Glo Luminescent Cell Viability assay purchased from Promega (Charbonnières, France), according to the manufacturer's instructions. Briefly, untreated and treated HepaRG cells were first incubated with the CellTiter-Glo reagent for 10 min at 37°C. Cells were then transferred in opaque-walled 96-well plates, and the luminescent signal was quantified using the POLARstar Omega microplate reader (BMG Labtech). Results were expressed in comparison to steatotic untreated cells.

### 2.13. Statistical Analysis

All results are expressed as mean ± SEM (standard error of mean). Comparisons between groups were performed using either two-way ANOVA followed by a post hoc Bonferroni test, one-way ANOVA followed by a post hoc Newman-Keuls test, or a *t*-test. All statistical analyses were performed using GraphPad Prism 5 software (GraphPad Software, San Diego, CA, USA).

## 3. Results

### 3.1. Lipid and Triglyceride Accumulation

**Figure 1:**
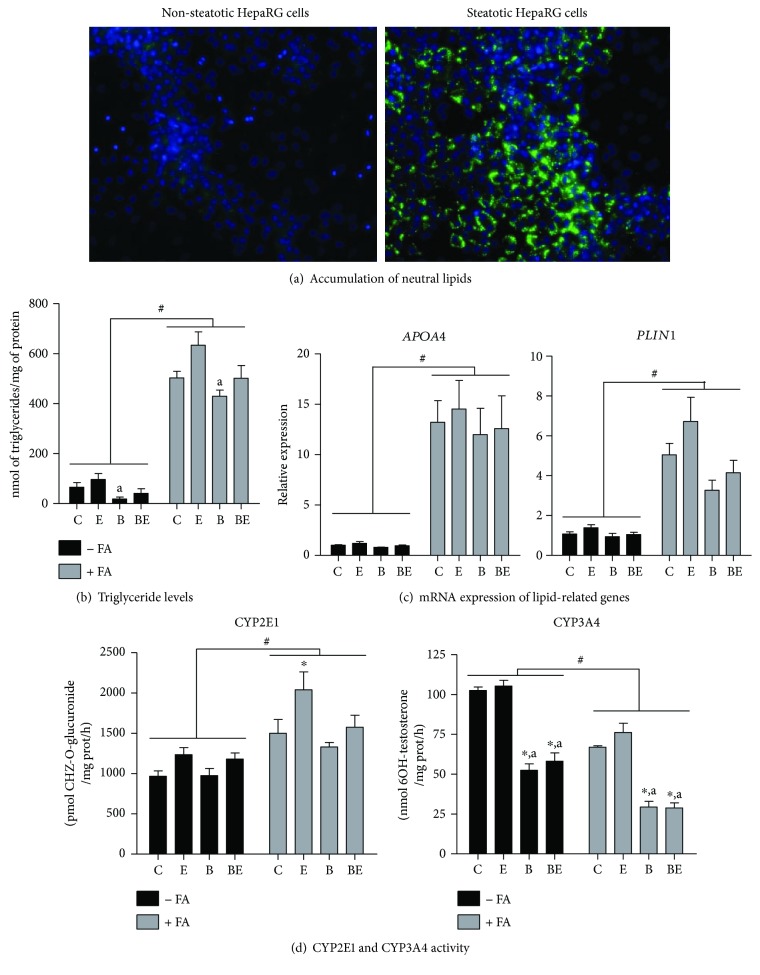
Fatty acid-overloaded HepaRG cells as a NAFL model. Steatosis was induced in HepaRG cells as described in Material and Methods. Nonsteatotic (−FA, black bars) and steatotic (+FA, grey bars) HepaRG cells were untreated (c) or treated with 25 mM ethanol (e), 2.5 *μ*M B[a]P (b), or a combination of both toxicants (BE) for 14 days. (a) Assessment of neutral lipid accumulation with Nile red at the end of the fatty acid treatment. (b) Triglyceride levels in the different groups of treatment. (c) mRNA expression of the lipid-related genes *APOA4* and *PLIN1* in the different groups of treatment. (d) CYP2E1 and CYP3A4 activity in the different groups of treatments. Results are means ± SEM for 4 (b and d) or 3 (c) independent cultures. ^#^Significantly different from nonsteatotic cells. ^∗^Significantly different from untreated nonsteatotic or steatotic HepaRG cells. ^a^Significantly different from nonsteatotic or steatotic HepaRG cells treated with ethanol only.

In a first series of experiments, the accumulation of neutral lipids was assessed in HepaRG cells overloaded or not with stearic and oleic acids. As expected, accumulation of neutral lipids and triglycerides were marked in fatty acid-overloaded HepaRG cells whereas these features were virtually absent in control cells (Figures 1(a) and 1(b)), in keeping with our recent investigations [14]. However, triglyceride levels were slightly but significantly lower in nonsteatotic and steatotic cells treated with B[a]P compared to cells treated with ethanol (Figure 1(b)). We also looked at the mRNA expression of apolipoprotein A4 (*APOA4*) and perilipin 1 (*PLIN1*) because these lipid-related genes are consistently overexpressed whenever steatosis occurs, including in the context of NAFLD [18, 30, 31]. In agreement with these reports, both *PLIN1* and *APOA4* were highly expressed in fatty acid-overloaded HepaRG cells (Figure 1(c)). Interestingly, changes of *PLIN1* and *APOA4* expression (Figure 1(c)) mostly paralleled those observed for triglyceride levels (Figure 1(b)). Finally, it is noteworthy that our cell model of NAFL was characterized by increased CYP2E1 activity and reduced CYP3A4 activity (Figure 1(d)), consistent with different clinical and experimental investigations [9, 32]. As expected [33], ethanol exposure also induced an increase in CYP2E1 activity, an effect that was more marked in steatotic cells (Figure 1(d)).

### 3.2. Mitochondrial Dysfunction

**Figure 2:**
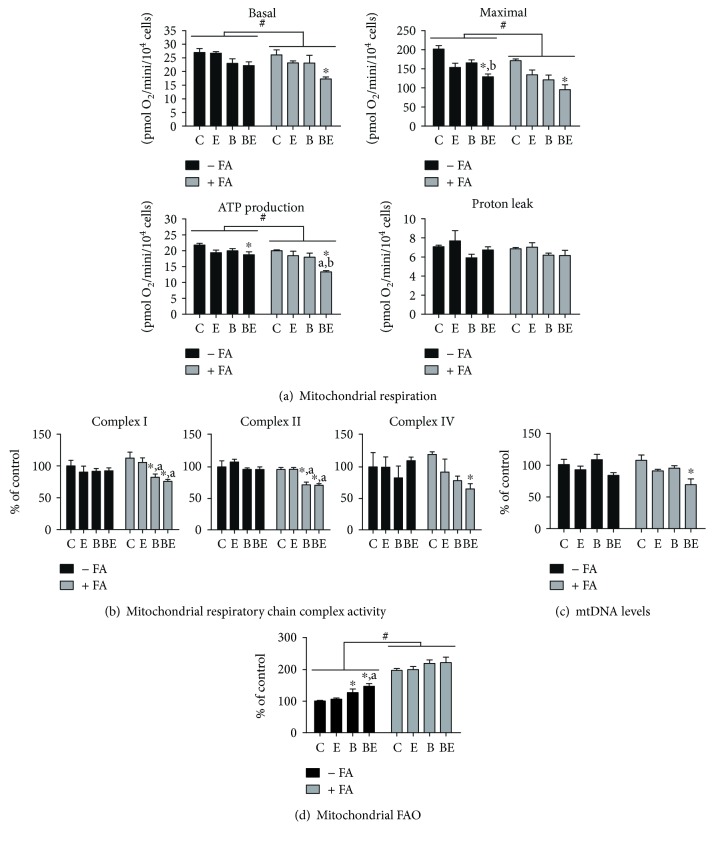
Mitochondrial effects of ethanol and B[a]P in nonsteatotic and steatotic HepaRG cells. Nonsteatotic (−FA, black bars) and steatotic (+FA, grey bars) HepaRG cells were untreated (c) or treated with 25 mM ethanol (e), 2.5 *μ*M B[a]P (b), or a combination of both toxicants (BE) for 14 days. (a) Parameters of mitochondrial respiration provided by the XF Cell Seahorse Mito Stress Test profile: basal respiration, maximal respiration, ATP production, and proton leak. (b) Activities of the MRC complexes I, II (SDH), and IV. (c) mtDNA levels. (d) mitochondrial FAO. Results are means ± SEM for 4 (a, b, and c) or 8 (d) independent cultures. ^#^Significantly different from nonsteatotic HepaRG cells. ^∗^Significantly different from untreated nonsteatotic or steatotic HepaRG cells. ^a^Significantly different from nonsteatotic or steatotic HepaRG cells treated with ethanol. ^b^Significantly different from nonsteatotic or steatotic HepaRG cells treated with B[a]P.

The effects of B[a]P, ethanol, and B[a]P/ethanol coexposure on different parameters pertaining to mitochondrial function were next assessed. Basal and maximal mitochondrial respiration as well as respiration linked to ATP production were decreased by B[a]P/ethanol coexposure, and this effect was significantly stronger in steatotic HepaRG cells (Figure 2(a)). Hence, the lowest rates of oxygen consumption was observed in steatotic HepaRG cells exposed to B[a]P plus ethanol (Figure 2(a)). In keeping with these results, activity of the MRC complexes I, II (SDH), and IV (Figure 2(b)) and mtDNA levels (Figure 2(c)) were the lowest in steatotic HepaRG cells exposed to B[a]P plus ethanol. However, reduced mtDNA levels were not associated with oxidative alterations of the mitochondrial genome assessed by long-range PCR (Supplementary Figure 2). Notably, SDH activity is independent of mtDNA since all SDH subunits are encoded only by nuclear DNA, in contrast to the other MRC complexes [34]. Thus, lower SDH activity in steatotic HepaRG cells exposed to B[a]P/ethanol (Figure 2(b)) could not be explained by reduced mtDNA levels (Figure 2(c)). Rather, it might be secondary to oxidative modifications of this enzyme, as previously reported [35, 36]. Mitochondrial FAO was also measured in the different conditions of treatment. Basal mitochondrial FAO was significantly enhanced in steatotic HepaRG cells compared with nonsteatotic HepaRG cells (Figure 2(d)), in agreement with clinical and experimental investigations performed in NAFLD [4, 15, 37, 38]. B[a]P and B[a]P/ethanol coexposure increased mitochondrial FAO, although this effect was statistically significant only in nonsteatotic HepaRG cells (Figure 2(d)). Nonetheless, the highest rate of mitochondrial FAO was observed in steatotic HepaRG cells exposed to B[a]P plus ethanol (Figure 2(d)).

We performed additional investigations in order to understand why mitochondrial FAO was enhanced by B[a]P in nonsteatotic HepaRG cells. PPAR*α* is a key transcription factor positively regulating the expression of different genes involved in FAO, in particular at the mitochondrial level [39]. Some investigations reported that AhR activation can increase PPAR*α* expression and activity in liver and hepatic cells [40, 41], although another study found an opposite result [42]. Thus, we determined whether B[a]P increased the mRNA expression of PPAR*α* (*PPARA*) and of 10 prototypical PPAR*α* target genes involved in mitochondrial FAO, namely, *CPT1A*, *CPT2*, *ACADVL*, *ACADM*, *ACADS*, *ACAA2*, *ETFDH*, *HADHA*, *HADHB*, and *SLC25A20* [39]. To do so, we took advantage of our transcriptomic analysis (GSE102536) recently performed in the same experimental model [14]. However, the expression of these genes was not significantly enhanced by B[a]P in nonsteatotic HepaRG cells (Supplementary Figure 3). Hence, more investigations are needed to determine the exact mechanism whereby B[a]P can enhance mitochondrial FAO. Finally, it was noteworthy that 6 out of the 10 PPAR*α* target genes presented higher expression in steatotic HepaRG cells (Supplementary Figure 3), which might explain why mitochondrial FAO was significantly increased in these cells (Figure 2(d)).

### 3.3. ROS Generation, Apoptosis, and Necrosis

**Figure 3:**
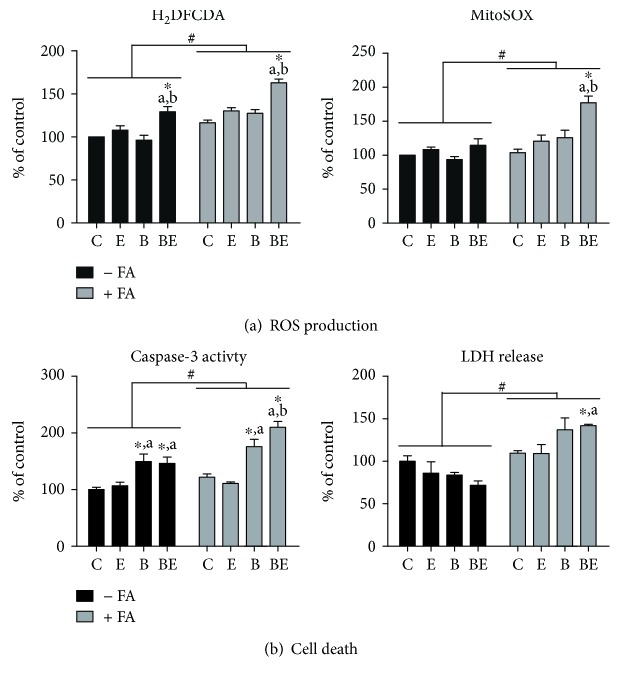
Effects of ethanol and B[a]P on ROS production and caspase-3 activity in nonsteatotic and steatotic HepaRG cells. Nonsteatotic (−FA, black bars) and steatotic (+FA, grey bars) HepaRG cells were untreated (c) or treated with 25 mM ethanol (e), 2.5 *μ*M B[a]P (b), or a combination of both toxicants (BE) for 14 days. (a) ROS production assessed with the H_2_DCFDA and MitoSOX Red dyes. (b) Cell death as assessed by cellular caspase-3 activity and LDH release in the culture medium. Results are means ± SEM for 6 (a) and 4 (b) independent cultures. ^#^Significantly different from nonsteatotic HepaRG cells. ^∗^Significantly different from untreated nonsteatotic or steatotic HepaRG cells. ^a^Significantly different from nonsteatotic or steatotic HepaRG cells treated with ethanol. ^b^Significantly different from nonsteatotic or steatotic HepaRG cells treated with B[a]P.

ROS were measured in this study with two different probes, namely, H_2_DCFDA and MitoSOX. Interestingly, both probes showed that the highest ROS production was observed in steatotic HepaRG cells exposed to B[a]P plus ethanol (Figure 3(a)). The mRNA expression of *SOD2* (manganese superoxide dismutase), a key enzyme involved in mitochondrial ROS detoxification [4], was unchanged in steatotic HepaRG cells coexposed to B[a]P/ethanol (data not shown). Because mitochondrial dysfunction and ROS overproduction can lead to apoptosis [43, 44] and necrosis [23, 24], caspase-3 activity and LDH release were assessed in the different conditions of treatment. Notably, the highest caspase-3 activity was observed in steatotic HepaRG cells exposed to B[a]P plus ethanol (Figure 3(b)). In addition, LDH release was the highest in steatotic HepaRG cells treated with B[a]P and ethanol, whereas no LDH release was observed in nonsteatotic cells treated with these compounds (Figure 3(b)). Thus, these results suggested that both apoptosis and necrosis occurred in steatotic HepaRG cells coexposed to B[a]P/ethanol.

### 3.4. Effects of the Antioxidant Tempol on Mitochondrial Dysfunction and ROS Production

**Figure 4:**
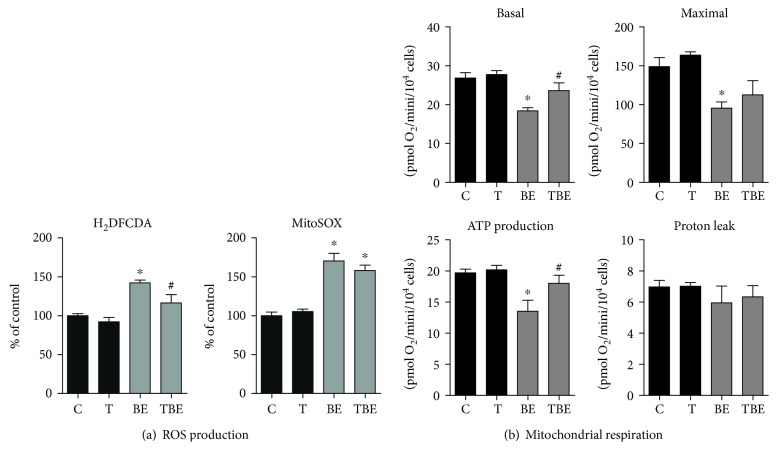
Effects of the antioxidant Tempol on ROS production and mitochondrial respiration in steatotic HepaRG cells exposed or not to ethanol and B[a]P. Steatotic HepaRG cells were untreated (c) or treated with 25 *μ*M Tempol (T), 2.5 *μ*M B[a]P, and 25 mM ethanol (BE) or a combination of the three compounds (TBE) for 14 days. (a) ROS production assessed with the H_2_DCFDA and MitoSOX Red dyes. (b) Parameters of mitochondrial respiration provided by the XF Cell Seahorse Mito Stress Test profile: basal respiration, maximal respiration, ATP production, and proton leak. Results are means ± SEM for 5 (a) and 3 (b) independent cultures. ^∗^Significantly different from untreated HepaRG cells. ^#^Significantly different from HepaRG cells treated with B[a]P and ethanol.

In a next series of investigations, we determined whether mitochondrial dysfunction and ROS overproduction induced by B[a]P/ethanol coexposure in steatotic HepaRG cells could be prevented by an antioxidant treatment. Preliminary investigations were first performed in order to screen different antioxidants (i.e., thiourea, vitamin E, MitoTEMPO, and Tempol) for their ability to reduce B[a]P/ethanol-induced cytotoxicity, as assessed by ATP levels. However, only Tempol (25 *μ*M) was found to be somewhat effective (Supplementary Figure 4A), whereas the other antioxidants were found to be either inefficient (MitoTEMPO) or even cytotoxic (thiourea and vitamin E) in our experimental conditions (Supplementary Figure 4B). Tempol slightly but significantly prevented ROS overproduction detected with H_2_DCFDA, but this preventive effect was not observed with MitoSOX (Figure 4(a)). Nonetheless, Tempol partially prevented B[a]P/ethanol-induced reduction of both basal mitochondrial respiration and respiration linked to ATP production in steatotic HepaRG cells, whereas its preventive effect was lesser regarding maximal respiration (Figure 4(b)). Thus, Tempol might have protected more efficiently the loss of OXPHOS-linked ATP production than the impairment of FCCP-driven maximal electron transfer induced by B[a]P/ethanol coexposure. In keeping with this assumption, B[a]P/ethanol-induced reduction of ATP levels (16%) was halved by Tempol (Supplementary Figure 4A). Altogether, these results suggested that mitochondrial dysfunction induced by B[a]P/ethanol coexposure in steatotic HepaRG cells was, at least in part, secondary to ROS overproduction.

### 3.5. Effects of the AhR Ligand ITE on ROS Production and Mitochondrial Dysfunction

**Figure 5:**
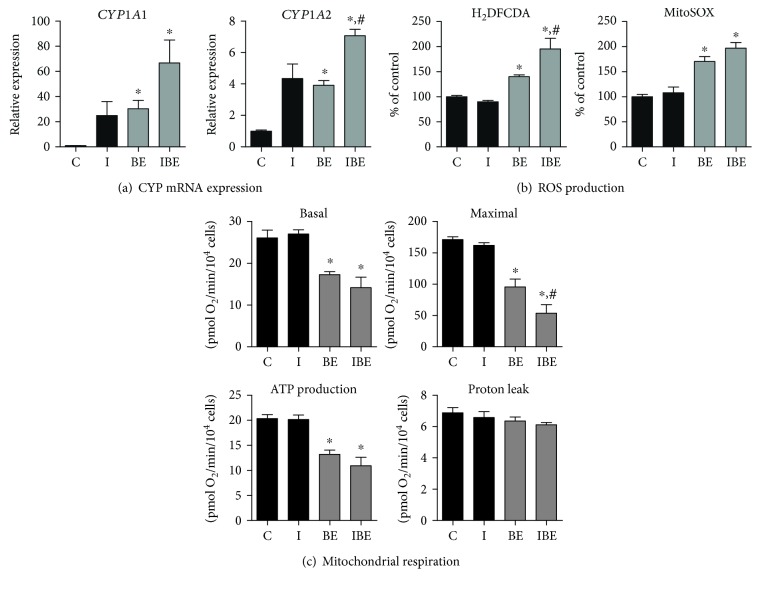
Effects of the AhR ligand ITE on CYP expression, ROS production, and mitochondrial respiration in steatotic HepaRG cells exposed or not to ethanol and B[a]P. Steatotic HepaRG cells were untreated (c) or treated with 25 *μ*M ITE (I), 2.5 *μ*M B[a]P, and 25 mM ethanol (BE) or a combination of the three compounds (IBE) for 14 days. (a) CYP1A1 and CYP1A2 mRNA expression. (b) ROS production assessed with the H_2_DCFDA and MitoSOX Red dyes. (c) Parameters of mitochondrial respiration provided by the XF Cell Seahorse Mito Stress Test profile: basal respiration, maximal respiration, ATP production, and proton leak. Results are means ± SEM for 3 (a), 5 (b), or 4 (c) independent cultures. ^∗^Significantly different from untreated HepaRG cells. ^#^Significantly different from HepaRG cells treated with B[a]P and ethanol.

Conversely to the aforementioned experiments, we wished to determine whether ROS overproduction and mitochondrial dysfunction induced by B[a]P/ethanol coexposure in steatotic HepaRG cells could be aggravated by reinforcing AhR activation with a cotreatment with ITE (25 *μ*M), a strong AhR ligand [45]. In preliminary experiments, we first verified that ITE did not induce cytotoxicity in steatotic HepaRG cells (data not shown). We also checked in these cells that ITE was able to further enhance the mRNA expression of CYP1A1 and CYP1A2 induced by B[a]P/ethanol coexposure (Figure 5(a)). In these conditions, ITE significantly increased B[a]P/ethanol-induced ROS production detected with H_2_DCFDA, whereas such exacerbation was not significant with MitoSOX (Figure 5(b)). Nevertheless, ITE further impaired B[a]P/ethanol-induced mitochondrial respiration, although this aggravating effect was significant only for the maximal respiration (Figure 5(c)). These results suggested that ROS overproduction and mitochondrial dysfunction induced by B[a]P/ethanol coexposure in steatotic HepaRG cells might be, at least in part, dependent on AhR activation. Finally, it was noteworthy that ITE by itself did not induce ROS production (Figure 5(b)) and did not impair mitochondrial respiration (Figure 5(c)) in steatotic HepaRG cells.

### 3.6. Effects of AhR Knockdown

Since AhR might play a role in ROS overproduction and mitochondrial dysfunction induced by B[a]P/ethanol coexposure in steatotic HepaRG cells, further investigations were performed in mut*AhR* HepaRG cells in which AhR expression was knocked down using the CRISPR/Cas9 technology.

**Figure 6:**
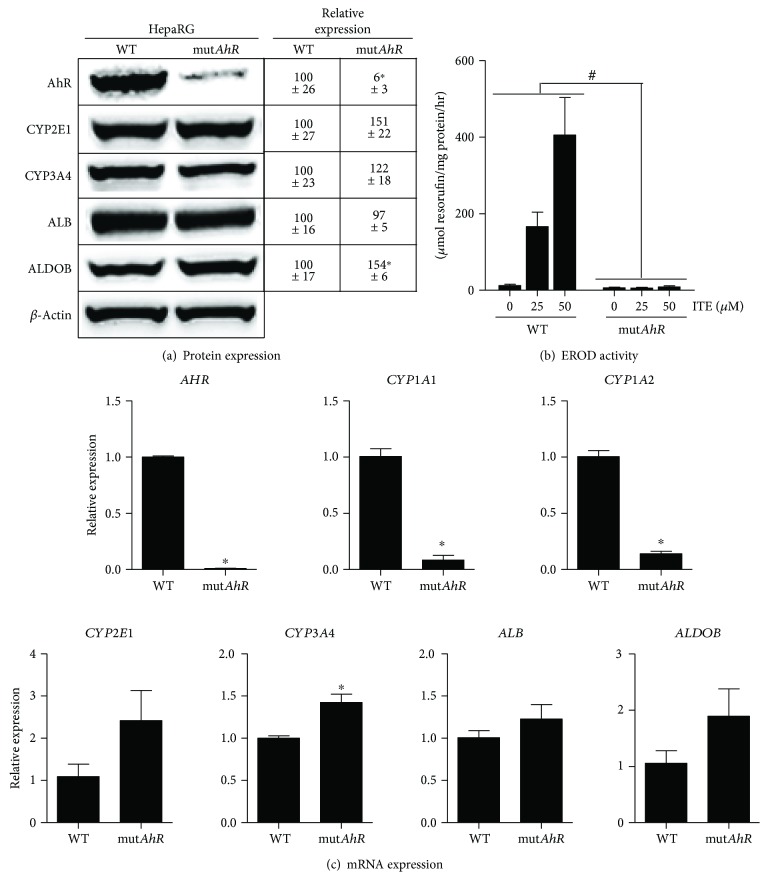
Characterization of the AhR-deficient HepaRG cells. HepaRG cells lacking functional AhR (mut*AhR*) were obtained using the CRISPR/Cas9 system as described in Materials and Methods and compared with wild-type (WT) HepaRG cells. Investigations were carried out in nonsteatotic condition. (a) Protein expression of AhR, CYP2E1, CYP3A4, albumin, aldolase B, and *β*-actin. Numbers mentioned in the table are means ± SEM of the *β*-actin-normalized expression of each protein for 3 independent cultures. (b) EROD activity in cells treated with 0, 25, or 50 *μ*M ITE for 48 hours. (c) mRNA expression of *AHR*, *CYP1A1*, *CYP1A2*, *CYP2E1*, *CYP3A4*, *ALB*, and *ALDOB*. Results are means ± SEM for 4 (b) or 3 (c) independent cultures. ^#,∗^Significantly different from WT HepaRG cells.

First, wild-type (WT) and AhR-deficient HepaRG cells were characterized in nonsteatotic condition (Figure 6). Compared to WT HepaRG cells, mut*AhR* HepaRG cells expressed very low levels of the AhR protein but presented normal (or subnormal) levels of CYP2E1, CYP3A4, albumin, and aldolase B (Figure 6(a)), which are specifically expressed in differentiated HepaRG cells [26]. Importantly, the mut*AhR* HepaRG cells were fully irresponsive to the dose-dependent effect of ITE on EROD activity (Figure 6(b)), thus indicating that the remaining low expression of AhR was unable to activate the expression of CYP1A1/2 and CYP1B1. The basal mRNA expression of *AHR*, *CYP1A1*, *CYP1A2*, *CYP2E1*, *CYP3A4*, albumin (*ALB*), and aldolase B (*ALDOB*) was also determined. *AHR* mRNA expression was barely detectable in the mut*AhR* HepaRG cells, whereas *CYP1A1* and *CYP1A2* mRNA expression was strongly decreased in these cells (Figure 6(c)). mRNA expression of *CYP2E1*, *ALB*, and *ALDOB* was not significantly different between WT and mut*AhR* HepaRG cells. Finally, mRNA expression of *CYP3A4* was significantly enhanced in mut*AhR* HepaRG cells (Figure 6(c)), consistent with recent investigations reporting a negative regulation of *CYP3A4* expression by AhR [46].

**Figure 7:**
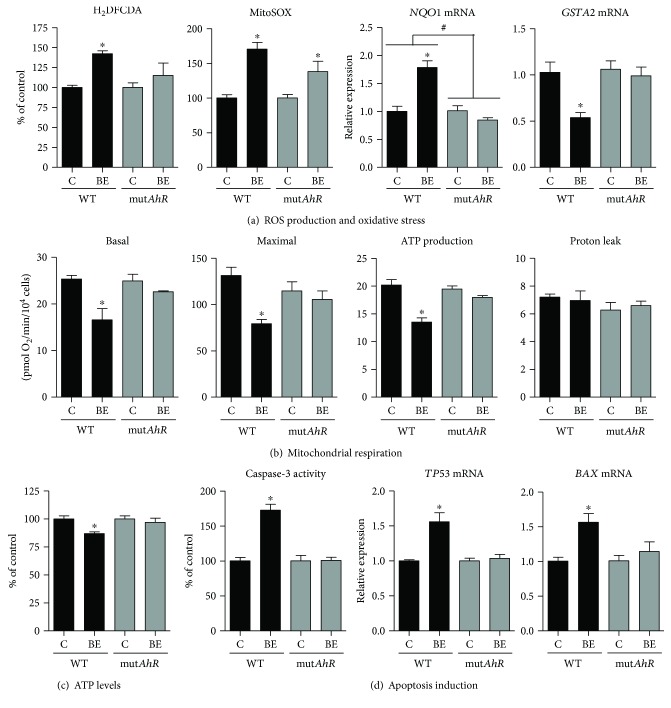
Effects of ethanol and B[a]P coexposure on ROS production, mitochondrial respiration, and apoptosis in steatotic wild-type and AhR-deficient HepaRG cells. Steatotic wild-type (WT) and AhR-deficient (mut*AhR*) HepaRG cells were untreated (c) or treated with a combination of 2.5 *μ*M B[a]P and 25 mM ethanol (BE) for 14 days. (a) ROS production assessed with the H_2_DCFDA and MitoSOX Red dyes and mRNA expression of *NQO1* and *GSTA2*. (b) Parameters of mitochondrial respiration provided by the XF Cell Seahorse Mito Stress Test profile: basal respiration, maximal respiration, ATP production, and proton leak. (c) ATP levels. (d) Caspase-3 activity and mRNA expression of *TP53* and *BAX*. Results are means ± SEM for 4 independent cultures. ^#^Significantly different from WT HepaRG cells. ^∗^Significantly different from untreated WT or mut*AhR* HepaRG cells.

Next, WT and AhR-deficient HepaRG cells were investigated in steatotic condition (Figure 7). ROS generation, mitochondrial respiration, ATP levels, and apoptosis parameters were indeed assessed in steatotic WT and mut*AhR* HepaRG cells exposed or not to B[a]P/ethanol for 14 days. AhR deficiency was first verified at this time point by assessing *CYP1A1* and *CYP1A2* mRNA expression. Whereas this expression was barely detectable in mut*AhR* HepaRG cells exposed to B[a]P/ethanol, mRNA levels of *CYP1A1* and *CYP1A2* were, respectively, increased 17- and 35-fold in WT HepaRG cells exposed to these compounds (Supplementary Figure 5). In addition, mRNA expression of *AHR* was almost undetectable in untreated and treated mut*AhR* HepaRG cells (Supplementary Figure 5). ROS overproduction induced by B[a]P/ethanol coexposure was diminished in mut*AhR* cells compared with WT HepaRG cells (Figure 7(a)). Moreover, the significant changes in *NQO1* (NAD(P)H quinone dehydrogenase 1) and *GSTA2* (glutathione S-transferase A2) mRNA expression observed in WT HepaRG cells treated by B[a]P/ethanol were absent in mut*AhR* cells (Figure 7(a)). B[a]P/ethanol-induced reduction of basal and maximal mitochondrial respiration as well as respiration linked to ATP production in WT HepaRG cells was also absent in mut*AhR* cells (Figure 7(b)). In keeping with this result, ATP levels were not reduced in mut*AhR* cells exposed to B[a]P plus ethanol (Figure 7(c)). Increased caspase-3 activity and mRNA expression of the proapoptotic factors *TP53* (tumor protein p53), *BAX* (Bcl2-associated X protein), *PMAIP1* (NOXA), and *FAS* induced by B[a]P/ethanol coexposure in WT HepaRG cells were abolished in mut*AhR* cells (Figure 7(d) and Supplementary Figure 6).

### 3.7. Acute Effects of AhR Activation on Mitochondrial Respiration

**Figure 8:**
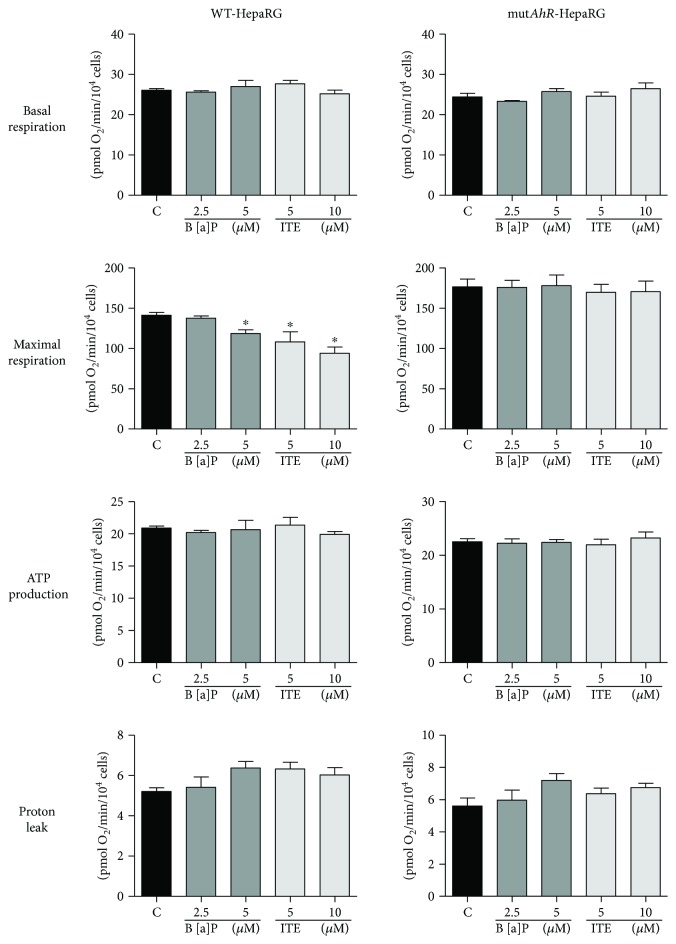
Acute effects of B[a]P and ITE on mitochondrial respiration in nonsteatotic AhR-deficient HepaRG cells. Nonsteatotic wild-type (WT) and AhR-deficient (mut*AhR*) HepaRG cells were untreated (C) or treated with B[a]P (2.5 or 5 *μ*M) or ITE (5 or 10 *μ*M) and mitochondrial respiration was assessed immediately afterwards. Parameters of mitochondrial respiration provided by the XF Cell Seahorse Mito Stress Test profile are basal respiration, maximal respiration, ATP production, and proton leak. Results are means ± SEM for 4 independent cultures. ^∗^Significantly different from untreated WT HepaRG cells.

In a last series of investigations, we wished to determine whether B[a]P (2.5 and 5 *μ*M) and ITE (5 and 10 *μ*M) could induce acute effects on mitochondrial respiration in both nonsteatotic WT and mut*AhR* HepaRG cells. B[a]P and ITE did not impair basal respiration and respiration linked to ATP production in both types of cells (Figure 8). In contrast, B[a]P and ITE reduced the maximal mitochondrial respiration in a concentration-dependent manner in WT HepaRG cells (Figure 8). Notably, these deleterious effects were not observed in mut*AhR* HepaRG cells (Figure 8). These results indicated that the mere activation of AhR can rapidly impair mitochondrial function, in agreement with recent investigations carried out with 2,3,7,8-tetrachlorodibenzo-p-dioxin (TCDD), another potent AhR activator [47].

## 4. Discussion

Because of the high prevalence of obesity, NAFLD is now the most common cause of chronic liver disease in numerous countries [1, 2]. This is particularly alarming for the public health because NAFL can progress to NASH, cirrhosis, and HCC [1, 2]. Different factors seem to favor the progression of NAFL to NASH including genetic polymorphisms [4, 5] and exposure to different xenobiotics such as drugs, alcohol, and environmental contaminants [6–9].

Recently, we showed in three different models of NAFL that B[a]P/ethanol coexposure promoted the occurrence of a steatohepatitis-like state characterized by cell death and increased expression of several inflammation markers [14]. Importantly, the deleterious effects induced by B[a]P plus ethanol were overall stronger than the respective effects observed for each compound [14]. Moreover, our data suggested that the steatohepatitis-like state induced by B[a]P/ethanol coexposure was favored by a profound effect of steatosis and ethanol on the expression of various XMEs leading to lower B[a]P detoxification and higher B[a]P bioactivation into toxic metabolites [14].

In the present study, we wished to determine whether mitochondrial dysfunction and ROS overproduction might play a role in the appearance of a steatohepatitis-like state in HepaRG cells overloaded with a mixture of stearic and oleic acids. Overall, the present data support such a role because mitochondrial dysfunction and ROS production were maximal in steatotic HepaRG cells coexposed to B[a]P/ethanol, whereas these deleterious events were less pronounced or absent in the other conditions of treatment (Figures 2 and 3). Notably, B[a]P/ethanol coexposure reduced MRC activity and enhanced mitochondrial FAO (Figure 2), which might particularly favor ROS overproduction as discussed later on. Moreover, B[a]P/ethanol-induced mitochondrial dysfunction might have favored mitochondrial ROS generation, as reported with other xenobiotics altering MRC activity [48–50]. Conversely, ROS overproduction might have played a role in B[a]P/ethanol-induced mitochondrial dysfunction since the antioxidant Tempol partially reversed the decrease in mitochondrial respiration (Figure 4). Nevertheless, whatever the involved mechanisms, ROS overproduction and mitochondrial dysfunction may have triggered necrosis and apoptosis in steatotic HepaRG cells treated with B[a]P/ethanol, although other mechanisms of cell death cannot be ruled out. For instance, previous studies performed in primary rat hepatocytes showed that B[a]P/ethanol coexposure was able to induce apoptosis *via* lysosomal membrane permeabilization [43].

There is increasing evidence that mitochondrial dysfunction and ROS overproduction play a major role in the progression of NAFL to NASH [4, 15, 51]. In the absence of xenobiotic exposure, mitochondrial dysfunction in NAFLD seems complex and may cover a wide array of metabolic alterations. In particular, NAFLD is associated with increased substrate oxidation through mitochondrial FAO and TCA cycle, a metabolic adaptation aiming at limiting lipid accumulation [4, 15, 37, 38]. However, this adaptation favors ROS overproduction because more electrons enter the MRC and leak from complexes I and III, the major sites of superoxide formation [4, 15, 52]. Importantly, this generation of mitochondrial ROS seems to trigger inflammation in NAFLD [15, 53, 54]. Moreover, investigations in another pathophysiological context indicate that mitochondrial ROS are involved in fibrosis initiation [55].

Mitochondrial dysfunction in NAFLD can also include an impairment of MRC activity, although this event seems to occur only when NASH develops [4, 56]. When MRC activity is reduced, ROS overproduction is expected to increase because the restricted flow of electron causes overreduction of complexes I and III and higher electron leakage from these MRC complexes [4, 37, 50]. Thus, mitochondrial ROS production and secondary oxidative stress is deemed to be higher in NASH compared to NAFL [4, 15]. Finally, a vicious circle could occur because more mitochondrial ROS formation will be able to further impair MRC activity either directly or indirectly *via* oxidative mtDNA damage [4, 44, 57]. Hence, in our experimental model, B[a]P/ethanol coexposure seems to have promoted the occurrence of a steatohepatitis-like state in steatotic HepaRG cells [14] *via* a mechanism akin to what occurs during the natural history of NAFLD. Indeed, this steatohepatitis-like state was associated with increased mitochondrial FAO (Figure 2(d)) while mitochondrial respiration, complex I, II (SDH), and IV activity, and mtDNA levels were significantly decreased (Figures 2(a)–2(c)). Importantly, these mitochondrial parameters were either unchanged, or less markedly impaired, in steatotic HepaRG cells treated with B[a]P alone or ethanol alone (Figure 2), which did not bring about such steatohepatitis-like state [14].

In this study, several investigations were performed in order to specify the role of AhR in B[a]P/ethanol-induced mitochondrial dysfunction and ROS production. The fact that ITE further increased ROS overproduction and further reduced mitochondrial respiration induced by B[a]P/ethanol coexposure (Figure 5) strongly suggested that AhR activation might be involved in these deleterious effects. This hypothesis was confirmed with our experiments carried out in AhR-deficient (mut*AhR*) HepaRG cells since lesser ROS production and no mitochondrial dysfunction were observed when these cells were coexposed to B[a]P/ethanol (Figures 7(a) and 7(b)). Lastly, normal ATP levels and the lack of apoptotic markers observed in mut*AhR* HepaRG cells treated with B[a]P/ethanol (Figures 7(c) and 7(d)) indicated that cell death was fully dependent on AhR activation.

Little is known regarding the mechanisms whereby B[a]P can impair MRC activity and mitochondrial respiration. B[a]P was reported to impair MRC activity through a mechanism that could involve the generation of CYP-derived toxic B[a]P metabolites and ROS [58]. These harmful derivatives could directly damage MRC components but could also induce mtDNA alterations able to alter mtDNA replication [58]. Indeed, B[a]P reactive metabolites are able to form covalent adducts to mtDNA [59, 60] and ROS can induce mtDNA abasic sites and strand breaks [34, 61]. However, it is noteworthy that B[a]P-induced MRC impairment and mtDNA reduction might have been aggravated by ethanol by different mechanisms, as discussed later on. Lastly, B[a]P-induced changes in mitochondrial matrix pH might also be involved. Indeed, we recently observed in B[a]P-treated hepatic epithelial cells that decreases in MRC complex II activity and oxygen consumption were related to matrix acidification [62].

Although CYP-derived toxic B[a]P metabolites and ROS could be involved in mitochondrial dysfunction, one cannot exclude the possibility that the mere activation of AhR could also impair MRC activity. Indeed, in this study, acute treatment with B[a]P (5 *μ*M) and ITE (5 or 10 *μ*M) induced a rapid (i.e., within the first hour) reduction of mitochondrial respiration in HepaRG cells (Figure 8). This mitochondrial effect seems transitory because no mitochondrial dysfunction was observed in HepaRG cells 24 hours after the last treatment (i.e., after the day 13 of treatment) with 25 *μ*M ITE (Figure 5). Interestingly, previous investigations showed that AhR is targeted to mitochondria and can interact with mitochondrial proteins such as ATP5*α*1, a subunit of ATP synthase [47, 63]. Moreover, one of these studies performed in two mouse hepatoma cell lines showed that TCDD was able to rapidly decrease mitochondrial respiration in AhR-expressing cells while this effect was almost abolished in AhR-deficient cells [47]. Thus, it is tempting to speculate that the mere ligand binding to the putative mitochondrial AhR could rapidly lead to its interaction with one or several key factor(s) involved in MRC activity and induce transient mitochondrial dysfunction. Because B[a]P and ITE acutely reduced FCCP-driven respiration but not respiration linked to ATP production (Figure 8), acute AhR activation in our experimental conditions might only impair maximal electron transfer but not OXPHOS-linked ATP synthesis per se.

In this study, a significant increase in mitochondrial FAO was observed in nonsteatotic HepaRG cells treated with B[a]P alone or with B[a]P/ethanol (Figure 2(d)). However, these effects were less substantial in steatotic HepaRG cells, most probably because mitochondrial FAO was already enhanced in the presence of fat accumulation. Previous investigations in chick embryos also reported that B[a]P was able to increase mitochondrial FAO, possibly *via* the generation of an oxygenated metabolite [64]. Further investigations will be requested in order to determine whether stimulation of mitochondrial FAO is specific to B[a]P or can also be induced by other AhR ligands.

Ethanol could have aggravated B[a]P-induced MRC impairment and mtDNA content reduction in steatotic HepaRG cells by at least two different mechanisms. First, CYP2E1-dependent generation of ROS and toxic metabolites (e.g., acetaldehyde and hydroxyethyl radical) can impair MRC activity. This can be caused either *via* direct alteration of key MRC components such as complex IV (cytochrome *c* oxidase) and ATP synthase or secondary to the presence of mtDNA damage leading to reduced mtDNA copy number [34, 61, 65, 66]. Interestingly, CYP2E1 activity was increased in steatotic HepaRG cells (Figure 1(d)), similar to what occurs in NAFLD patients [67–69]. Second, ethanol treatment in steatotic HepaRG cells could have favored the formation of B[a]P metabolites able to impair directly or indirectly MRC activity. Indeed, our recent study showed that the steatohepatitis-like state in HepaRG cells was characterized by a strong downregulation of various XMEs that was associated with lesser B[a]P detoxification and higher B[a]P bioactivation [14]. However, comparison of the data between the eight different culture conditions indicated that the effects observed in steatotic HepaRG cells treated with B[a]P/ethanol were mostly attributable to fatty acid overload rather than to ethanol itself [14].

In this study, ROS generation was assessed with the H_2_DCFDA and MitoSOX Red dyes. These fluorescent probes allow the detection of cellular hydrogen peroxide and mitochondrial superoxide anion, respectively [21, 22]. However, these probes are not fully specific and could detect other types of ROS or oxidants [21, 22]. For instance, H_2_DCFDA could detect hydroxyl radical and nitrogen dioxide [21, 22]. These points might explain why different results were obtained in some investigations (e.g., condition BE in nonsteatotic cells in Figure 3(a)), although there was generally a good concordance of the results obtained with H_2_DCFDA and MitoSOX Red.

Several parameters pertaining to mitochondrial function, ROS generation, and cell death were not significantly changed in nonsteatotic cells treated for 14 days with B[a]P and ethanol (Figures 2 and 3). However, we cannot exclude the possibility that higher concentrations of B[a]P and ethanol and/or longer exposure to these compounds might have induced stronger mitochondrial dysfunction and more robust ROS production in these cells. The occurrence of such marked effects in nonsteatotic cells might thus allow to study the role of AhR in B[a]P/ethanol-induced mitochondrial dysfunction and ROS production in the absence of lipid overload.

## 5. Conclusions

The main results of this study allow providing the following conclusions:

- Coexposure to B[a]P and ethanol impairs mitochondrial function, increases ROS generation, and induces cell death by apoptosis and necrosis in fatty acid-overloaded HepaRG cells, whereas these deleterious effects were less marked or absent in nonsteatotic cells.
- These results, along with our data recently obtained in the same model of NAFL [14], strongly suggest that B[a]P/ethanol-induced mitochondrial dysfunction and ROS generation are involved in the occurrence of a steatohepatitis-like state in steatotic HepaRG cells.
- B[a]P/ethanol-induced impairment of mitochondrial respiration, ROS production, and apoptosis appears to be dependent on AhR activation.
- Although CYP-derived toxic B[a]P metabolites and ROS could be involved in mitochondrial dysfunction, the mere activation of AhR could also induce such deleterious effect.

## Data Availability

The data used to support the findings of this study are available from the corresponding author upon request.

## Supplementary Materials

Supplementary Materials The supplementary materials include one table and 6 figures. Supplementary Table 1: primer sequences (5′ to 3′) used for real-time PCR experiments. Supplementary Figure 1: protocol of HepaRG cell culture and treatments. Supplementary Figure 2: long-range PCR for the detection of oxidative mtDNA alterations in nonsteatotic and steatotic HepaRG cells treated with ethanol and B[a]P. Supplementary Figure 3: mRNA expression of PPAR*α* and of 10 PPAR*α* target genes in nonsteatotic and steatotic HepaRG cells treated with ethanol and B[a]P. Supplementary Figure 4: effects of different antioxidant compounds on B[a]P/ethanol-induced ATP level reduction in steatotic HepaRG cells. Supplementary Figure 5: mRNA expression of AHR, CYP1A1, and CYP1A2 in steatotic wild-type and AhR-deficient HepaRG cells coexposed to ethanol and B[a]P. Supplementary Figure 6: mRNA expression of PMAIP1 (NOXA) and FAS in steatotic wild-type and AhR-deficient HepaRG cells coexposed to ethanol and B[a]P.
