# Supplementary material for: Possible Involvement of Mitochondrial Dysfunction and Oxidative Stress in a Cellular Model of NAFLD Progression Induced by Benzo[a]pyrene/Ethanol CoExposure
Source: Oxid Med Cell Longev. 2018 Jul 26;2018:4396403. doi: 10.1155/2018/4396403 (PMC6083493; doi:10.1155/2018/4396403)
Supplement: Supplementary Materials — The supplementary materials include one table and 6 figures. Supplementary Table 1: primer sequences (5′ to 3′) used for real-time PCR experiments. Supplementary Figure 1: protocol of HepaRG cell culture and treatments. Supplementary Figure 2: long-range PCR for the detection of oxidative mtDNA alterations in nonsteatotic and steatotic HepaRG cells treated with ethanol and B[a]P. Supplementary Figure 3: mRNA expression of PPARα and of 10 PPARα target genes in nonsteatotic and steatotic HepaRG cells treated with ethanol and B[a]P. Supplementary Figure 4: effects of different antioxidant compounds on B[a]P/ethanol-induced ATP level reduction in steatotic HepaRG cells. Supplementary Figure 5: mRNA expression of AHR, CYP1A1, and CYP1A2 in steatotic wild-type and AhR-deficient HepaRG cells coexposed to ethanol and B[a]P. Supplementary Figure 6: mRNA expression of PMAIP1 (NOXA) and FAS in steatotic wild-type and AhR-deficient HepaRG cells coexposed to ethanol and B[a]P. [file 4396403.f1.pdf]

Supplementary Materials

Possible involvement of mitochondrial dysfunction and oxidative stress in a cellular model of NAFLD progression induced by benzo[a]pyrene/ethanol co-exposure

Simon Bucher, Dounia Le Guillou, Julien Allard, Grégory Pinon, Karima Begriche, Arnaud Tête, Odile Sergent, Dominique Lagadic-Gossmann, Bernard Fromenty

Supplementary Table 1: Primers sequences (5’ to 3’) used for real-time PCR experiments.

Supplementary Figure 1: Protocol of HepaRG cell culture and treatments.

Supplementary Figure 2: Long-range PCR for the detection of oxidative mtDNA alterations in non-steatotic and steatotic HepaRG cells treated with ethanol and B[a]P.

Supplementary Figure 3: mRNA expression of PPARα and of 10 PPARα target genes in non-steatotic and steatotic HepaRG cells treated with ethanol and B[a]P.

Supplementary Figure 4: Effects of different antioxidant compounds on B[a]P/ethanol-induced ATP level reduction in steatotic HepaRG cells.

Supplementary Figure 5: mRNA expression of *AHR*, *CYP1A1* and *CYP1A2* in steatotic wild-type and AhR-deficient HepaRG cells co-exposed to ethanol and B[a]P.

Supplementary Figure 6: mRNA expression of *PMAIP1* (*NOXA*) and *FAS* in steatotic wild-type and AhR-deficient HepaRG cells co-exposed to ethanol and B[a]P.

Gene	Accession Number	Forward primer	Reverse primer
AHR	NM_001621.4	cttcagccaccatccatactt	cctttggcatcacaaccaatag
ALB	NM_000477.5	tgcttgaatgtgctgatgacagg	aaggcaagtcagcaggcatctcatc
ALDOB	NM_000035.3	gcatctgtcagcagaatgga	tagacagcagccaggacctt
APOA4	NM_000482.3	cagtgtggcaagaaactcct	gtagtcccacatcaccgtg
BAX	NM_001291429.1	ggagctgcagaggatgattg	agttgaagttgccgtcagaa
CYP1A1	NM_000499.4	tcttccttcgtccccttcac	acaccttgtcgatagcacca
CYP1A2	NM_000761.4	gacatctttggagcaggatttg	ccttctggatcttcctctgtatc
CYP2E1	NM_000773.3	ttgaagcctctcgttgaccc	cgtggtgggatacagcca
CYP3A4	NM_017460.5	cttcatccaatggactgcataaat	tcccaagtataacactctacacagacaa
FAS	NM_000043.5	tcctcaattccaatcccttg	gcatctggaccctcctacct
NQO1	NM_000903.2	acgtccttcaactatgccatg	ttacctgtgatgtcctttctgg
PLIN1	NM_002666.4	tggtcctcatgatcctcctc	gttgtcgatgtcccggaatt
PMAIP1	NM_021127.2	ttcggtcactacacaacgtaaa	agtaacgcccaacaggaac
TBP	NM_003194.4	tgatggacgcctttatcctc	ccacgacctgatccaattc
TP53	NM_001276696.1	cttccatttgctttgtcccg	catctcccaaacatccctcac

Supplementary Table 1: Primers sequences (5’ to 3’) used for real-time PCR experiments.

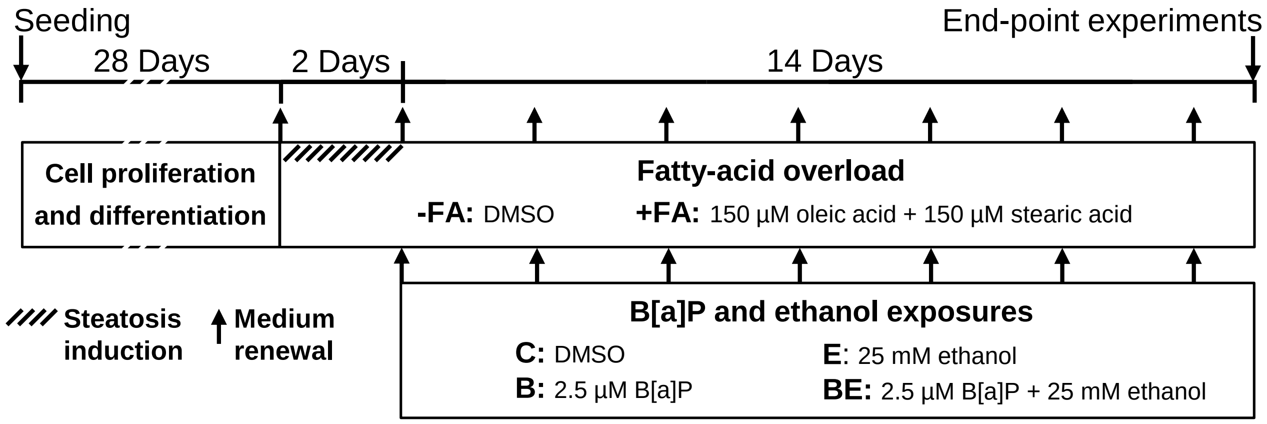

Supplementary Figure 1: Protocol of HepaRG cell culture and treatments.

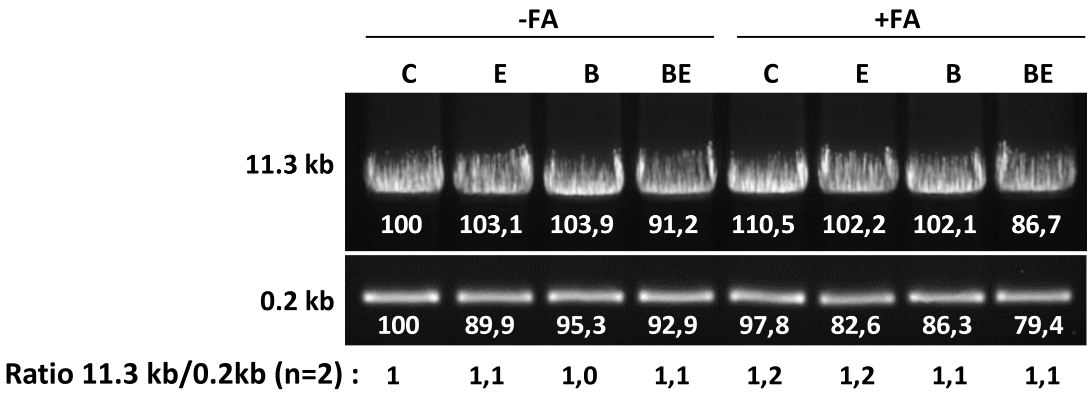

Supplementary Figure 2: Long-range PCR for the detection of oxidative mtDNA alterations in non-steatotic and steatotic HepaRG cells treated with ethanol and B[a]P. Non-steatotic (-FA) and steatotic (+FA) HepaRG cells were untreated (C) or treated with 25 mM ethanol (E), 2.5 µM B[a]P (B), or a combination of both toxicants (BE) for 14 days. At the end of the treatments, total DNA was extracted and 2 different fragments (199 and 11,326 bp) of the mitochondrial DNA (mtDNA) were amplified using Promega GoTaq G2 Hot Start Colorless Master Mix and Promega GoTaq Long PCR Master Mix, respectively. Forward and reverse primers used for the amplification of the short mtDNA fragment were 5'-AATCCTACCTCCATCGCTAACC-3' and 5'-GTGTGGTCGGGTGTGTTATTATTC-3', respectively. Forward primer used for the amplification of the long mtDNA fragment was 5'-GAGCCCGGTAATCGCATAAA-3' whereas reverse primer was the same as the one used for the short mtDNA fragment. The data are representative of 2 different experiments. The lack of reduced amplification of the 11.3 kb amplicon indicates the absence of oxidative mtDNA lesions able to impair DNA synthesis, such as abasic sites and strand breaks [55, 61].

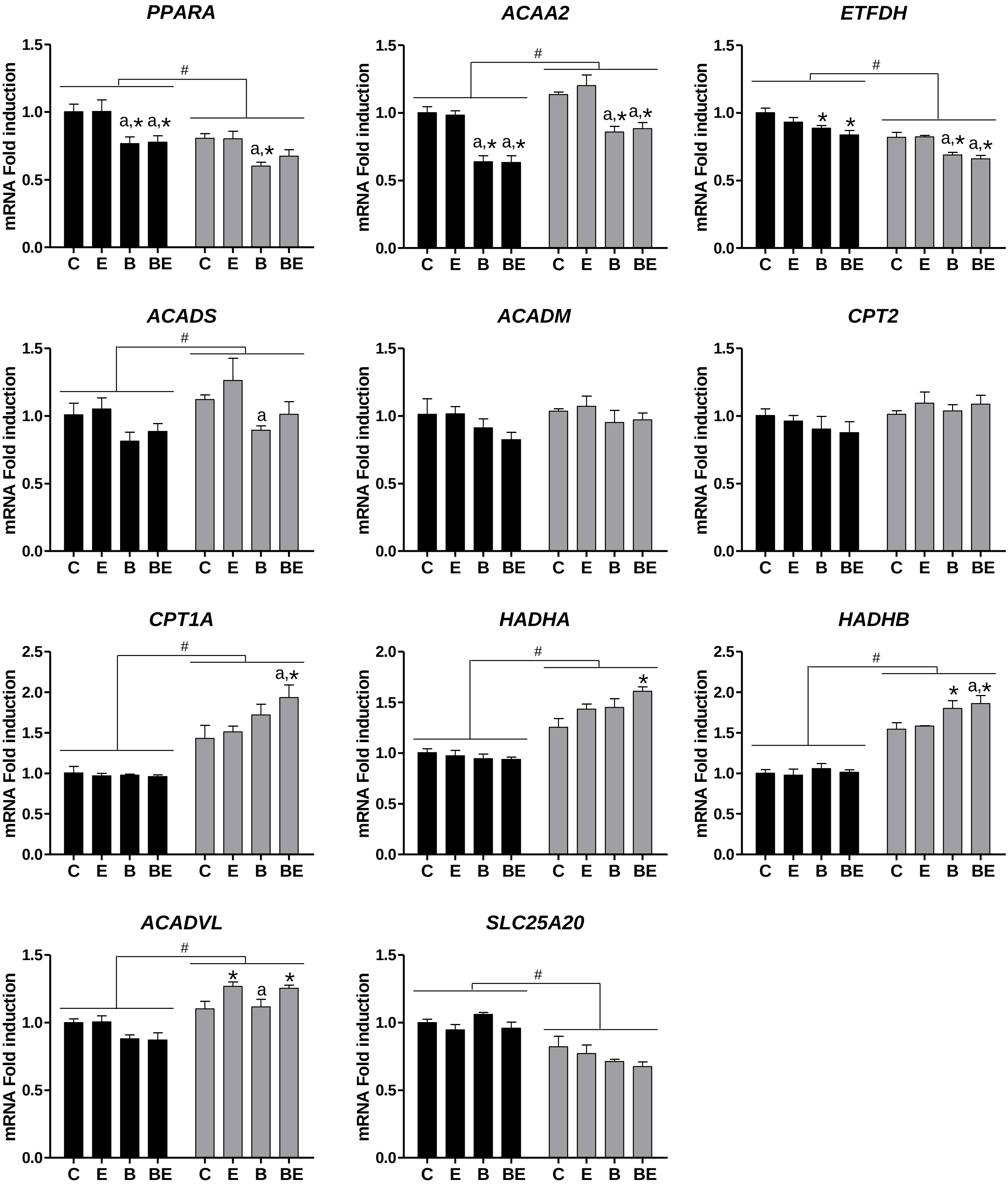

Supplementary Figure 3: mRNA expression of PPARα and of 10 PPARα target genes in non-steatotic and steatotic HepaRG cells treated with ethanol and B[a]P. Non-steatotic (-FA, black bars) and steatotic (+FA, grey bars) HepaRG cells were untreated (C) or treated with 25 mM ethanol (E), 2.5 µM B[a]P (B), or a combination of both toxicants (BE) for 14 days. mRNA expression of PPARα (*PPARA*) and 10 PPARα target genes encoding mitochondrial FAO enzymes was determined at the end of the treatment (microarray dataset GSE102536 [14]). Results are means ± SEM for 4 independent cultures. [#]Significantly different from non-steatotic HepaRG cells. [*]Significantly different from untreated non-steatotic or steatotic HepaRG cells. [a]Significantly different from non-steatotic or steatotic HepaRG cells treated with ethanol.

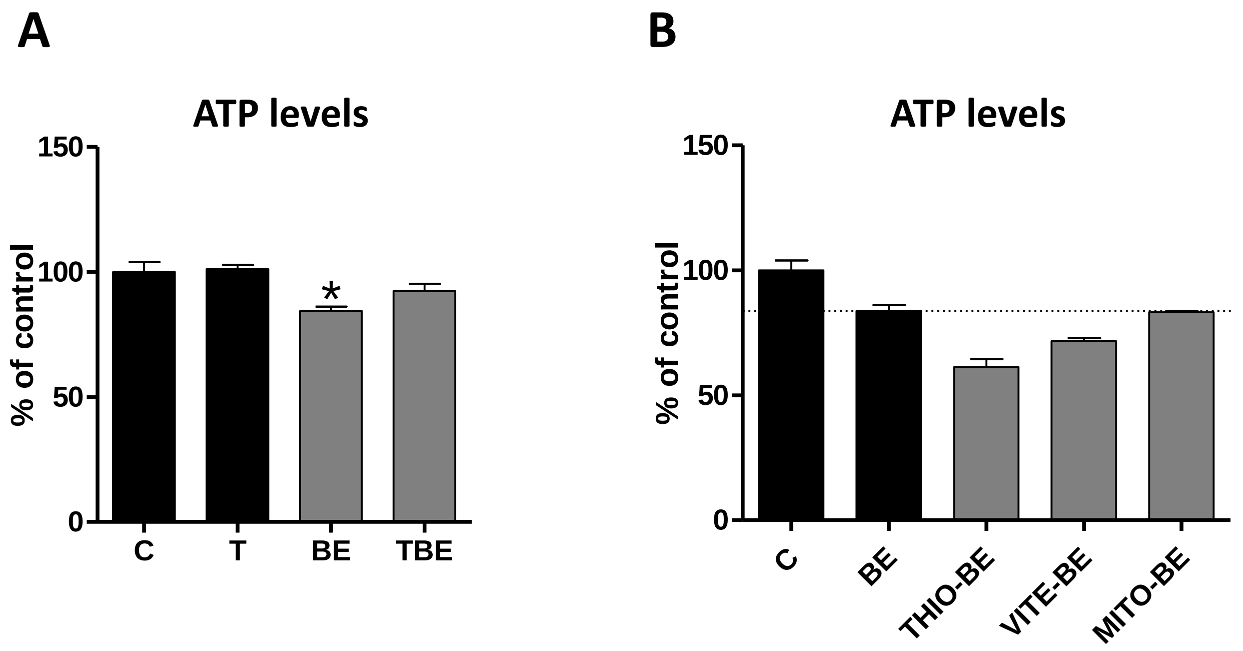

Supplementary Figure 4: Effects of different antioxidant compounds on B[a]P/ethanol-induced ATP level reduction in steatotic HepaRG cells. A. Steatotic HepaRG cells were untreated (C) or treated for 14 days with 25 µM Tempol (T), a combination of 2.5 µM B[a]P and 25 mM ethanol (BE), or with BE in association with Tempol (TBE). **B.** Preliminary investigations performed in steatotic HepaRG cells, which were untreated (C) or treated for 14 days with a combination of 2.5 µM B[a]P and 25 mM ethanol (BE), or with BE in association with 10 mM thiourea (THIO-BE), 100 µM vitamin E (VITE-BE) and 25 µM MitoTempo (MITO-BE). Results are means ± SEM for 4 (Panel A) and 2 (Panel B) independent cultures. *Significantly different from untreated HepaRG cells.

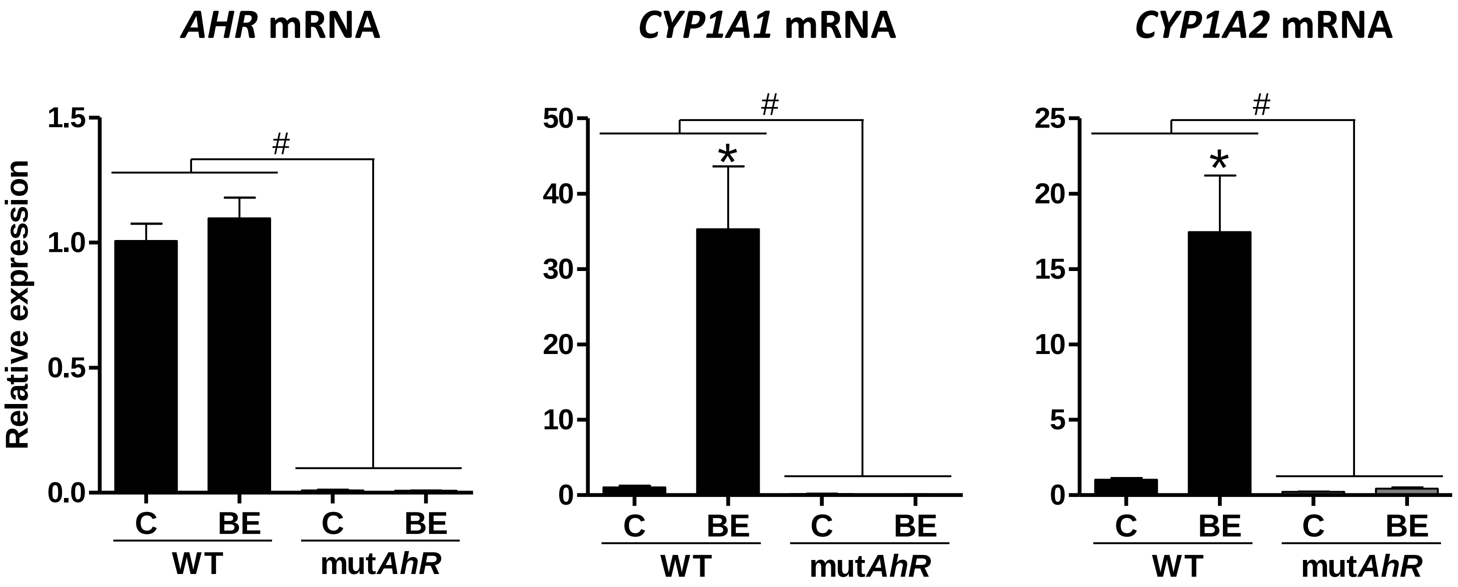

Supplementary Figure 5: mRNA expression of *AHR, CYP1A1* and *CYP1A2* in steatotic wild-type and AhR-deficient HepaRG cells co-exposed to ethanol and B[a]P. Steatotic wild-type (WT) and AhR-deficient (mut*AhR*) HepaRG cells were untreated (C) or treated with a combination of 2.5 µM B[a]P and 25 mM ethanol (BE) for 14 days. mRNA expression of *AHR, CYP1A1* and *CYP1A2* was determined at the end of the treatment. Results are means ± SEM for 4 independent cultures. [#]Significantly different from WT HepaRG cells [*]Significantly different from untreated WT HepaRG cells.

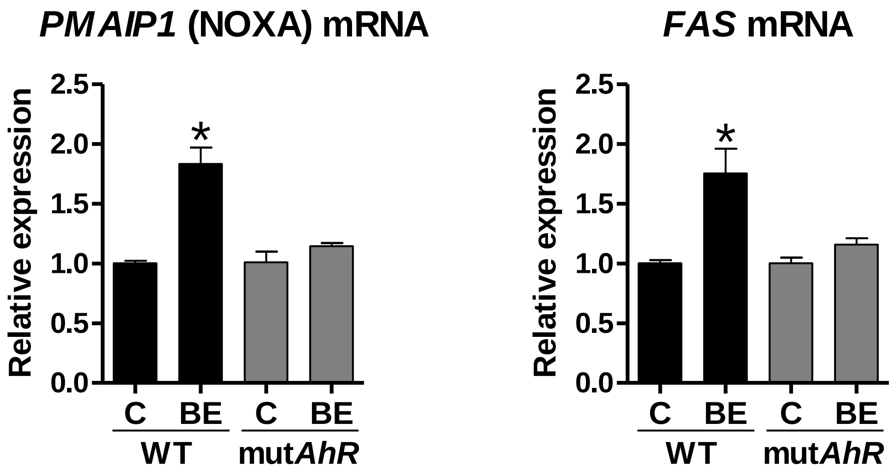

Supplementary Figure 6: mRNA expression of *PMAIP1* (*NOXA*) and *FAS* in steatotic wild-type and AhR-deficient HepaRG cells co-exposed to ethanol and B[a]P. Steatotic wild-type (WT) and AhR-deficient (mutAhR) HepaRG cells were untreated (C) or treated with a combination of 2.5 µM B[a]P and 25 mM ethanol (BE) for 14 days. mRNA expression of *PMAIP1* (*NOXA*) and *FAS* was determined at the end of the treatment. Results are means ± SEM for 4 independent cultures. *Significantly different from untreated WT HepaRG cells.
